# Punctate Hyperfluorescent Spots on Indocyanine Green Angiography in Eyes with Central Serous Chorioretinopathy and Patient Demographics

**DOI:** 10.3390/jcm15010249

**Published:** 2025-12-29

**Authors:** Setsuko Kawakami, Mariko Sasaki, Yoshihiro Wakabayashi, Tsuyoshi Mizusawa, Hideki Mori, Hiroshi Goto, Tsutomu Yasukawa

**Affiliations:** 1 Department of Ophthalmology, Tokyo Medical University, 6-7-1 Nishishinjuku, Shinjuku-ku, Tokyo 160-0023, Japan; 2 Department of Ophthalmology, Keio University, 35 Shinanomachi, Shinjuku-ku, Tokyo 160-8582, Japan; 3 Department of Ophthalmology, National Hospital Organization Tokyo Medical Center, 2-5-1 Higashigaoka, Meguro-ku, Tokyo 152-8902, Japan; 4 Department of Ophthalmology & Visual Science, Graduate School of Medical Sciences, Nagoya City University, 1 Kawasumi, Mizuho-cho, Mizuho-ku, Nagoya 467-8602, Japan

**Keywords:** central serous chorioretinopathy, choroidal vascular hyperpermeability, pachychoroid, pachydrusen, punctate hyperfluorescent spots

## Abstract

**Purpose:** To investigate the clinical significance of punctate hyperfluorescent spots (PHS) on indocyanine green angiography (ICGA) in patients with central serous chorioretinopathy (CSCR). **Methods:** In this retrospective study, 87 eyes of 87 patients diagnosed with CSCR through comprehensive multimodal imaging were analyzed. Eyes with a cluster of five or more PHS on ICGA were classified as PHS (+), and eyes with four or fewer as PHS (−). Clinical parameters, including age, blood pressure, visual acuity, and imaging features such as choroidal venous dilation, choroidal vascular hyperpermeability (CVH), pachydrusen, and the status of PHS in the CSCR-unaffected fellow eyes, were compared between the two groups. Logistic regression analysis was performed to identify independent factors associated with PHS (+) status. **Results:** The PHS (+) group consisted of 63 (72.4%) eyes and the PHS (−) group 24 (27.6%) eyes. In the PHS (+) group, patients were significantly older (*p* = 0.031) and had higher systolic blood pressure (*p* = 0.030) and worse best-corrected visual acuity (*p* = 0.040) than those in the PHS (−) group. Notably, the PHS (+) group showed a significantly higher prevalence of PHS clusters in the fellow eye than the PHS (−) group (81.7% vs. 34.8%, *p* < 0.001). In univariate analysis, age (*p* = 0.045), venous dilation (*p* = 0.041), CVH (*p* = 0.034), and PHS clusters in the fellow eye (*p* < 0.001) were significantly associated with PHS clusters in the study eye. In multivariate analysis adjusted for multiple confounders, venous dilation (*p* = 0.026) and the presence of cluster PHS in the fellow eye (*p* = 0.001) remained significantly associated, and CVH tended to be significant (*p* = 0.089). **Conclusions:** PHS clusters are a frequent finding in eyes with CSCR, associated with venous dilation, CVH, and PHS in the CSCR-unaffected fellow eye. These conditions may predispose eyes to pachychoroid diseases such as CSCR.

## 1. Introduction

Central serous chorioretinopathy (CSCR) is characterized by serous retinal detachment (SRD) resulting from disruption of the outer blood–retinal barrier. The pathogenesis of CSCR is not fully understood, but several contributing factors have been identified. Choroidal vascular hyperpermeability (CVH) is a hallmark of CSCR, possibly associated with thickened choroid (also known as pachychoroid) and SRD through dysfunction of retinal pigment epithelium (RPE) [1,2,3]. In addition, CSCR is considered to be affected by systemic factors such as stress and corticosteroid exposure [1,4].

CSCR is generally classified into acute or chronic types. Acute CSCR exhibits more typical findings than chronic CSCR, including active leakage on fluorescein angiography (FA) and pachychoroid, and often resolves spontaneously. In contrast, chronic CSCR shows persistent SRD and more varied findings [3]. While the precise pathophysiology of CSCR remains elusive, recent advances in multimodal imaging have provided significant insights, particularly regarding the choroidal circulation and RPE [5]. Indocyanine green angiography (ICGA) is a valuable tool for differential diagnosis and assessing the condition of CSCR, depicting characteristic findings including choriocapillaris filling delay and dilated choroidal veins as well as CVH [6,7,8,9]. Additionally, punctate hyperfluorescent spots (PHS), observable in the middle and late phase of ICGA, have been observed in eyes with CSCR [10,11]. PHS are not only observed in CSCR but also in polypoidal choroidal vasculopathy (PCV). CSCR and PCV are considered to be in the same classification, that is, pachychoroid diseases characterized by dilated choroidal veins connecting to vortex veins in the Haller’s layer and thinning of the overlying choriocapillaris and Sattler’s layer [12,13,14,15,16]. PHS have been recently reported to be a finding preceding pachydrusen [11,16,17]. Nevertheless, the clinical significance of these findings remains unclear.

In this study, we investigated the relationship of PHS to other clinical findings on multi-modal imaging and patient demographics to explore the clinical significance of PHS in the pathophysiology of CSCR.

## 2. Patients and Methods

### 2.1. Ethics Statement

The study was conducted in accordance with the tenets of the Declaration of Helsinki and was approved by the Institutional Review Board of Tokyo Medical University (approval number: T2023-0035). All the subjects provided written informed consent.

### 2.2. Study Design

This retrospective study was conducted at a single center. We reviewed the medical records of patients diagnosed with CSCR at Tokyo Medical University between March 2018 and December 2023. Patients underwent ophthalmic examinations, including best corrected visual acuity (BCVA) measurement, slit-lamp biomicroscopy, fundus examination, fundus photography, infrared image, fundus autofluorescence (FAF), FA, ICGA with the field of view set to 30° × 30° (Spectralis HRA + OCT; Heidelberg Engineering, Heidelberg, Germany), swept-source optical coherence tomography (SS-OCT), and OCT angiography (DRI-OCT Triton; Topcon Corp, Tokyo, Japan).

Eyes with a history of intravitreal injection of anti-vascular endothelial growth factor drug, intravitreal or sub-Tenon’s injection of triamcinolone acetonide, systemic steroid, retinal photocoagulation, photodynamic therapy, or vitreous surgery were excluded.

Visual acuity was measured using a decimal visual acuity chart and converted to the logarithm of the minimal angle resolution (logMAR) for statistical analysis.

Foveal retinal thickness (FRT) and foveal choroidal thickness (FCT) were measured using an SS-OCT device. FRT was defined as the vertical distance between the internal limiting membrane (ILM) and the ellipsoid zone (EZ) under the fovea. FCT was defined as the vertical distance between Bruch’s membrane and the interface of the choroid and the sclera under the fovea. FRT and FCT were measured manually on a B-scan image using the caliper measurement tool embedded in the OCT system. Measurements of FRT and FCT were performed by two individuals, SK and YW, and the average values were evaluated.

### 2.3. Diagnosis and Classification of CSCR

CSCR was diagnosed according to the following criteria, as reported previously [5]. Major criteria [which must fulfill the following (1) and (2) for a diagnosis of CSCR]: (1) presence or evidence of prior SRD detectable on OCT involving the posterior pole, unrelated to other disease entities including dome-shaped macula; inferior staphyloma; uveal effusion syndrome; inherited retinal diseases; adult-onset foveomacular vitelliform dystrophy; choroidal nevus, choroidal osteoma, and other intraocular tumors; Vogt–Koyanagi–Harada disease, posterior scleritis, and other intraocular inflammatory diseases; drug toxicity; myopic tractional maculopathy; diabetic retinopathy, retinal vein occlusion, and other retinal vascular diseases; optic pit maculopathy; neovascular age-related macular degeneration and other diseases with macular neovascularization; and systemic diseases such as hypertensive retinopathy and choroidopathy and hypertensive disorder of pregnancy; and (2) presence of RPE alteration detectable on fundus autofluorescence, OCT, or infrared imaging. Minor criteria [which must fulfill the following (1), (2), or (3)]: (1) CVH, detected as middle- to late-phase hyperfluorescent placoid areas on ICGA; (2) focal leakage points on FA; or (3) remarkable pachychoroid defined as 400 μm or longer of SCT in this study. We classified CSCR into simple and complex subtypes: eyes with RPE alterations of 2-disc area or smaller as simple CSCR, and eyes with RPE alterations larger than 2-disc areas as complex CSCR. The classification of simple versus complex CSCR was based on FAF. The area of RPE alteration was measured manually on FAF images obtained using the Spectralis HRA. The classification of CSCR as simple or complex was performed by two raters (SK and YW). Interobserver agreement was assessed using Cohen’s kappa coefficient. The kappa coefficient for the presence of complex CSCR was 0.893 (almost perfect agreement). Any discrepancies between raters were resolved through direct discussion, leading to a consensus. Each subtype was subdivided further into 3 groups as reported previously [5]: primary CSCR; first known episode of subretinal fluid (SRF), recurrent CSCR; presence of SRF with history or signs of previous SRF on FAF, and resolved CSCR; absence of SRF on OCT. Furthermore, the classification of acute and chronic was as follows: acute CSCR was defined as primary or resolved CSCR with subjective ocular symptoms such as central scotoma and metamorphopsia lasting less than 6 months, and chronic CSCR as recurrent CSCR or any CSCR with SRF or symptoms lasting 6 months or longer. All recurrent cases were included in the “chronic” CSCR group. In patients with bilateral CSCR, the eye with earlier symptom onset was selected as the CSCR-affected eye.

On ICGA, filling delay in the choroidal circulation was evaluated in the early phase, venous dilation was examined in the middle phase, and CVH was determined in the late phase (15 to 20 min after dye injection). PHS are hyperfluorescent spots observed in the middle to late phase of ICGA [11]. They were classified into two patterns: a solitary pattern defined as less than 5 PHS, and a cluster pattern as 5 or more PHS, according to the classification in a previous report [15]. We also investigated the status and findings of PHS in the CSCR-unaffected fellow eyes.

Pachydrusen was diagnosed when all of the following criteria were met, as reported previously [18]: (1) the drusen diameter was larger than 125 µm; (2) the outer contours of the drusen were irregular; (3) the drusen occurred in isolation or in groups of only a few, showing a scattered distribution over the posterior pole.

Choriocapillaris filling delay, venous dilation, CVH, PHS, and pachydrusen were independently evaluated by two retinal specialists (SK and YW). Discrepancies in viewpoints were resolved through direct discussion, leading to a consensus among the parties. We investigated whether there were differences in patient demographics as well as fundus, FA, and ICGA findings between patients with and without PHS clusters.

### 2.4. Statistical Analysis

Statistical analyses were performed using IBM SPSS Statistics Software version 29.0 (SPSS Inc., Chicago, IL, USA). Fisher’s exact test was used for analysis of categorical variables, and the Mann–Whitney U test was used for analysis of continuous variables. Univariate analyses were performed to detect factors related to the presence of cluster PHS in the CSCR-affected eyes. Multivariate logistic regression analysis was performed to detect factors independently associated with cluster PHS in the CSCR-affected eyes. A *p* value less than 0.05 was considered statistically significant.

Two independent raters measured FCT and FRT using OCT images. Interobserver reliability was assessed using the intraclass correlation coefficient, employing a single measurement, absolute agreement, two-way mixed-effects model [ICC (3,1)]. Interpretation of the ICC followed conventional benchmarks [19], where values < 0.50 indicate poor reliability, 0.50–0.75 indicate moderate reliability, 0.75–0.90 indicate good reliability, and ≥0.90 indicate excellent reliability. The interobserver reliability for FCT was excellent, with ICC (3,1) of 0.91 (confidence interval [CI]: 0.87–0.94). The FRT data did not follow a normal distribution, as tested by the Shapiro–Wilk test. Therefore, Spearman’s rank correlation coefficient was used to assess the association between the FRT values of the two examiners, yielding ρ of 0.80 (*p* < 0.001), indicating a strongly significant correlation.

A number of imaging parameters were independently evaluated by two individuals. Interobserver agreement for categorical variables was assessed using Cohen’s kappa coefficient. The results were interpreted according to Landis and Koch [19]: kappa coefficients ≥ 0.81, 0.61–0.80, 0.41–0.60, 0.21–0.40, and ≤0.20 indicate almost perfect, substantial, moderate, fair, and slight agreement, respectively. The kappa coefficient for the presence of cluster PHS was 1.000, filling delay 0.838, venous dilation 0.903, CVH 0.941, pachydrusen 0.850, and cluster PHS in the CSCR-unaffected fellow eye 0.944 (almost perfect agreement) [20].

## 3. Results

### 3.1. Clinical Characteristics of Patients with or Without PHS Clusters

FA and ICGA were performed in 165 patients with unilateral or bilateral CSCR. Among these patients, 87 patients who met the selection criteria and had data for the pre-determined analyses were enrolled in the study. In four patients with bilateral CSCR, the eye with earlier symptom onset was studied as the CSCR-affected eye. Hence, the present study was conducted in 87 patients by comprehensive multimodal imaging. The CSCR-affected eyes with PHS cluster on ICGA were classified into the PHS (+) group, and those without PHS clusters as the PHS (−) group.

**Figure 1:**
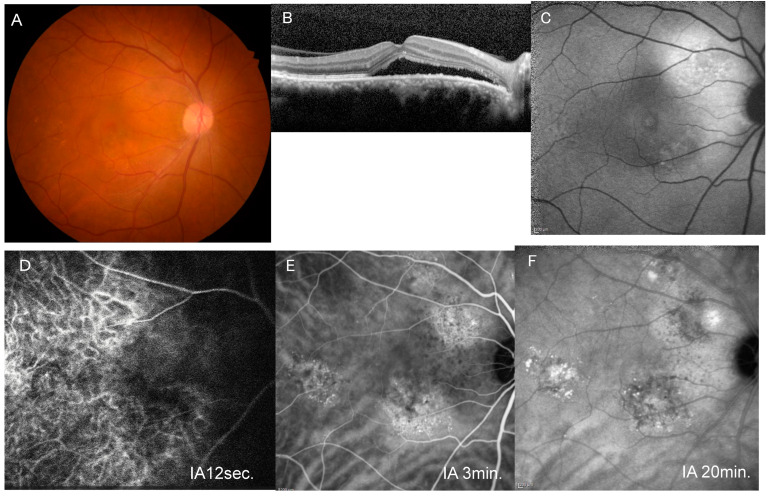
A representative case of unilateral central serous chorioretinopathy with cluster PHS. (**A**) Fundus photograph of the right eye shows pachydrusen temporal to the macula. (**B**) Horizontal image of optical coherence tomography through the fovea shows serous retinal detachment. (**C**) Blue-light fundus autofluorescence image shows an area with mixed hyper- and hypo-autofluorescence superotemporal to the disc. (**D**) Early-phase indocyanine green angiography (IA) image (12 s after dye injection) shows filling delay in the macular area. (**E**) Middle-phase IA image (3 min after injection) shows venous dilation and a cluster of punctate hyperfluorescent spots. (**F**) Late-phase IA (20 min after injection) image shows a hyperfluorescent area around the area with cluster PHS.

**Figure 2:**
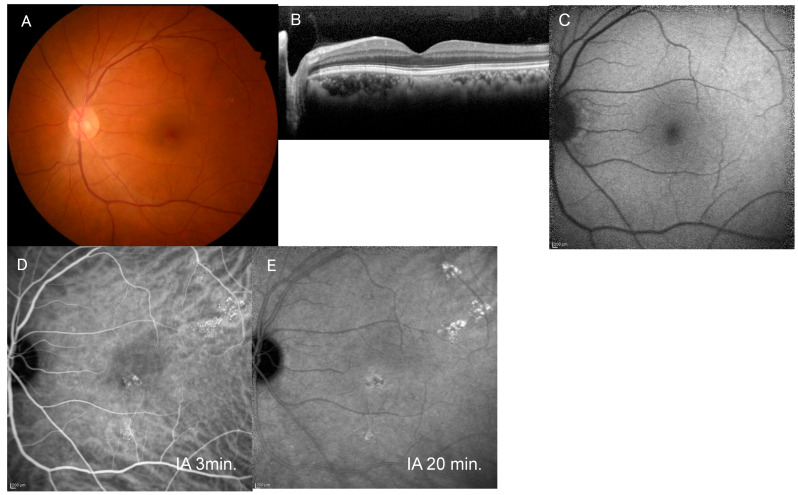
Images of the fellow eye in the case shown in Figure 1. (**A**) Fundus photograph of the left eye shows pachydrusen superotemporal to the macula. (**B**) Horizontal image of optical coherence tomography through the fovea shows no serous retinal detachment. (**C**) Blue-light fundus autofluorescence image shows no abnormal autofluorescence. (**D**) Mid-phase indocyanine green angiography (IA) image (3 min after injection) shows a cluster of punctate hyperfluorescent spots. (**E**) Late-phase IA (20 min after injection) image shows a slight hyperfluorescent area around the area with cluster PHS.

The clinical characteristics of the PHS (+) and PHS (−) groups are listed in Table 1. Of the 87 eyes, 63 eyes (72.4%) had cluster PHS (Figure 1 and Figure 2), while 19 eyes (21.8%) had solitary PHS. Five eyes (5.7%) had no PHS. In the PHS (+) group, the mean age was 54.2 ± 13.1, significantly older as compared with 47.9 ± 11.8 years in the PHS (−) group (*p* = 0.031). The PHS (+) group had higher systolic blood pressure (132 ± 17 vs. 125 ± 13 mmHg, *p* = 0.030) and poorer BCVA (0.09 ± 0.21 vs. 0.03 ± 0.26 in the LogMAR unit, *p* = 0.040). There were no significant differences in FCT (425 ± 109 vs. 383 ± 119 µm, *p* = 0.129), FRT (ILM-EZ) (174 ± 44 vs. 179 ± 38 µm, *p* = 0.333), proportion of complex CSCR (85.7% vs. 70.8%, *p* = 0.128), and proportion of chronic CSCR (49.2% vs. 45.8%, *p* = 0.814) between the two groups (Table 1).

### 3.2. Characteristics of Images of the CSCR-Affected Eyes with or Without Cluster PHS

Eyes in the PHS (+) group had apparently higher prevalence of venous dilation (92.0% vs. 75.0%, *p* = 0.064), CVH (81.0% vs. 58.3%, *p* = 0.051), and pachydrusen (38.1% vs. 20.8%, *p* = 0.203) than eyes in the PHS (−) group, although any differences reached no statistical significance (Table 1). However, the prevalence of cluster PHS in the CSCR-unaffected fellow eyes was significantly higher in the PHS (+) group than in the PHS (−) group (81.7% vs. 34.8%, *p* < 0.001).

Additionally, we conducted analysis separately for acute and chronic CSCR to investigate whether the association between PHS clusters and imaging features differed according to CSCR stage. No significant differences were observed between the PHS (+) and PHS (−) groups in acute CSCR or chronic CSCR for filling delay, venous dilation, CVH, and pachydrusen. Prevalence of cluster PHS in the CSCR-unaffected fellow eyes was significantly higher in the PHS (+) group than in the PHS (−) group in acute CSCR (81.3% vs. 23.1%, *p* < 0.001), while no significant difference was found in chronic CSCR (82.1% vs. 50.0%, *p* = 0.090) (Table 2).

### 3.3. Logistic Regression Analysis for Factors Associated with Cluster PHS

Univariate logistic regression analysis revealed that older age (*p* = 0.045; odds ratio [OR], 1.04; CI, 1.00–1.09), venous dilation (*p* = 0.041; OR, 3.87; CI, 1.06–14.18), CVH (*p* = 0.034; OR, 3.04; CI, 1.09–8.48), and presence of cluster PHS in the CSCR-unaffected fellow eyes (*p* < 0.001; OR, 8.35; CI, 2.84–24.57) were significantly associated with the presence of cluster PHS in the CSCR-affected eyes. Systolic blood pressure (*p* = 0.064; OR, 1.03; CI, 1.00–1.07), FCT (*p* = 0.119; OR, 1.00; 95% CI, 1.00–1.01), and complex CSCR (*p* = 0.116; OR, 2.47; CI, 0.80–7.63) showed borderline associations (Table 3).

We conducted multivariate logistic regression analysis, including variables showing significant or borderline association in univariate analysis as confounders (Table 3). Analysis adjusted for age and male sex (Model 1) identified the presence of cluster PHS in the fellow eye (*p* < 0.001; OR, 7.02; CI, 2.21–22.27) as the significant factor associated with cluster PHS in the CSCR-affected eye. Adjustment for age, male sex, systolic blood pressure, and foveal choroidal thickness (Model 2) identified venous dilation (*p* = 0.027; OR, 5.53, CI, 1.22–25.12) and presence of cluster PHS in the fellow eye (*p* = 0.001; OR, 8.47; CI, 2.37–30.26) as significant factors, while CVH tended to be significant (*p* = 0.093; OR, 2.88; CI, 0.84–9.87). Furthermore, adjustment for age, gender, and systolic blood pressure, foveal choroidal thickness, and complex CSCR (Model 3) also yielded similar results [venous dilation (*p* = 0.026; OR, 5.82, CI, 1.24–27.30), presence of cluster PHS in unaffected fellow eye (*p* = 0.001; OR, 9.68; CI, 2.45–38.29), CVH (*p* = 0.089; OR, 2.93; CI, 0.85–10.11)].

## 4. Discussion

In this study, we investigated 87 eyes of 87 patients with CSCR and evaluated the presence of cluster PHS in the CSCR-unaffected fellow eyes for the 83 eyes of 83 patients with unilateral CSCR. Previous studies have reported that PHS are frequently observed in the CSCR-unaffected fellow eyes [11,13]. Kamao et al. reported that, in patients with unilateral macular neovascularization (MNV), the presence of PHS in the fellow eye was associated with increased choroidal thickness and a higher prevalence of pachychoroid pigment epitheliopathy [21]. Therefore, they suggested that, among patients with unilateral MNV, those with bilateral PHS and those without PHS in either eye may represent distinct patient populations. However, in patients with CSCR, no studies have investigated whether the presence or absence of PHS in the affected eye influences the occurrence of PHS in the unaffected fellow eye.

Previous studies have demonstrated that choroidal vascular abnormalities detected by ICGA frequently persist in both eyes of patients with CSCR, even after resolution of serous retinal detachment, and may predispose to recurrence or fellow eye involvement [8,22]. In the present study, the prevalence of cluster PHS in the unaffected fellow eye was significantly higher in patients with cluster PHS in the CSCR-affected eye than in those without cluster PHS (81.7% vs. 34.8%, *p* < 0.001). This finding suggests that PHS may represent a predisposing factor rather than a secondary change resulting from CSCR. However, it is also possible that the CSCR-unaffected eyes may include undiagnosed CSCR cases, as these eyes have minimal or no symptoms. Because acute CSCR can resolve spontaneously, and previous episodes may not always be documented, it is difficult to rule out the possibility that PHS is a consequence of CSCR. Moreover, given the cross-sectional design of this study, a causal relationship between PHS and CSCR cannot be inferred. Nevertheless, even if PHS reflects a consequence rather than a predisposing factor, it may still have clinical value as a potential indicator of previous CSCR episodes.

Regarding PHS and CVH, Kim et al. reported a higher incidence of CVH in PCV eyes with PHS [14]. In our study, eyes with cluster PHS showed a trend of high prevalence of venous dilation and CVH, although the differences did not reach statistical significance. Univariate logistic regression analysis revealed venous dilation and CVH as factors associated with cluster PHS. Age, systolic blood pressure, complex CSCR, and foveal choroidal thickness were found to be significant or borderline significant in univariate analysis and were considered important confounding factors. In the multivariate analyses, venous dilation was significantly associated with cluster PHS after adjustment for age, male sex, systolic blood pressure, FCT, and complex CSCR.

Previous studies have reported that the presence of PHS did not affect FCT in eyes with both CSCR and PCV [13,14]. Consistent with these findings, our analysis revealed no significant difference in FCT between eyes with and without cluster PHS. However, Jirarattanasopa et al. [23] previously reported localized thickening at the sites with PHS, indicating that not only FCT but also choroidal thickness at other local areas should be assessed to detect subtle variations.

In the present study, CSCR subtypes (complex or not, chronic or not) were not associated with the presence of cluster PHS. Additionally, we conducted analysis separately for acute and chronic CSCR to investigate whether the association between PHS clusters and imaging features differed according to CSCR phase. No significant differences were observed between the PHS (+) and PHS (−) groups in acute CSCR or chronic CSCR for filling delay, venous dilation, CVH, and pachydrusen. Prevalence of cluster PHS in the CSCR-unaffected fellow eyes was significantly higher in the PHS (+) group than in the PHS (−) group in acute CSCR, while no significant difference was found in chronic CSCR (Table 2).

Localized hyperfluorescence on the middle phase of ICGA is a well-known feature of CSCR [24]. PHS often appear at the center of the hyperfluorescent area, which is expanded over time. Therefore, the origin of hyperfluorescence on the middle to late phase of ICGA may be close to the area with PHS [11]. Bousquet et al. [25] proposed that the middle phase hyperfluorescent plaques may reflect mild dysfunction of the RPE. If cluster PHS share a similar pathophysiological mechanism with the middle-phase hyperfluorescent plaques and represent an early finding of RPE alteration, this may explain why the presence of cluster PHS was not associated with CSCR subtypes classified spatiotemporally.

The pathogenesis of PHS remains uncertain; proposed mechanisms include leakage from tiny punctate sites within the inner choroid [11], late-phase staining of sub-RPE deposits such as forme fruste drusen, or a combination of both [14]. During ICGA, indocyanine green dye is predominantly bound to phospholipid in serum lipoproteins and circulates in blood, then gradually translocates onto paravascular lipid and plasma membrane, which is relevant to middle-phase and late-phase hyperfluorescence. The concept of pachydrusen has recently been introduced as a pachychoroid disease-associated finding [18]. Matsumoto et al. [17] reported the presence of pachydrusen in 27.2% of CSCR eyes, often co-localizing with PHS, which suggests a potential predictive relationship between PHS and pachydrusen development. Pachydrusen are sub-RPE deposits whose composition has been unclear [17]. In eyes with PCV and pachychoroid neovasculopathy, a significant correlation was detected between CVH and PHS as well as between PHS and pachydrusen [16]. Notably, new drusen emerged at sites previously occupied by PHS during a mean follow-up of 74.8 months in 21% of eyes [16]. We speculate that PHS reflect the staining of punctate lipid-rich deposits under the RPE and that further accumulation of serum-derived lipid accumulation may result in the formation of pachydrusen-like circinate hard exudate in diabetic macular edema. Thus, we advocate that pachydrusen can be named “circinate drusenoid exudate”.

Our study demonstrated a trend toward a higher prevalence of pachydrusen in eyes with cluster PHS, although the difference was not statistically significant. Kang et al. [16] reported a strong association between pachydrusen and both CVH and PHS in terms of their topographic and temporal distributions, indicating that cross-sectional studies, including ours, may have inherent limitations in fully clarifying these relationships. The cross-sectional design and limited sample size are acknowledged as limitations in this study. To clarify the pathogenesis and clinical significance of cluster PHS in eyes with CSCR and other pachychoroid diseases, further longitudinal studies of larger cohorts are warranted.

This article is a revised and expanded version of a paper titled “Clinical significance of punctate hyperfluorescent spots observed on indocyanine green angiography in eyes with central serous chorioretinopathy,” which was presented at the 24th Euretina congress, Barcelona Spain, on 19–22 September 2024 [26].

## 5. Conclusions

In eyes with CSCR, the presence of cluster PHS was associated with CVH. Cluster PHS were frequently observed in the fellow eyes of patients with unilateral CSCR. These findings suggest that cluster PHS may reflect a predisposing finding rather than a secondary manifestation of CSCR.

## Figures and Tables

**Table 1:** Clinical characteristics of patients having a CSCR-affected eye with or without cluster PHS.

	PHS (+) Group	PHS (−) Group	*p* Value
Number of eyes, *n* (%)	63 (72.4%)	24 (27.6%) no PHS, 5; solitary (<5 PHS), 19	
Male, *n* (%)	51 (81.0%)	20 (83.3%)	1.000
Age, years	54.2 ± 13.1	47.9 ± 11.8	0.031
Hypertension, *n* (%)	24 (38.1%)	4 (16.7%)	0.073
Diabetes, *n* (%)	7 (11.1%)	0	0.183
Systolic blood pressure, mmHg	132 ± 17	125 ± 13	0.030
Diastolic blood pressure, mmHg	80 ± 15	76 ± 10	0.118
Visual acuity, logMAR	0.09 ± 0.21	0.03 ± 0.26	0.040
Foveal choroidal thickness, µm	425 ± 109	383 ± 119	0.129
Foveal retinal thickness (ILM-EZ), µm	174 ± 44	179 ± 38	0.333
Simple CSCR, *n* (%)	9 (14.3%)	7 (29.2%)	
Complex CSCR, *n* (%)	54 (85.7%)	17 (70.8%)	0.128
Acute CSCR, *n* (%)	32 (50.8%)	13 (54.2%)	
Primary, *n*	31	13	
Resolved, *n*	1	0	
Chronic CSCR, *n* (%)	31 (49.2%)	11 (45.8%)	0.814
Primary, *n*	12	4	
Recurrent, *n*	16	7	
Resolved, *n*	3	0	
Filling delay, *n* (%)	55 (87.3%)	21 (87.5%)	1.000
Venous dilation, *n* (%)	58 (92.0%)	18 (75.0%)	0.064
Choroidal vascular hyperpermeability, *n* (%)	51 (81.0%)	14 (58.3%)	0.051
Pachydrusen, *n* (%)	24 (38.1%)	5 (20.8%)	0.203
CSCR unilateral	60	23	
Cluster PHS in CSCR-unaffected fellow eye, *n* (%)	49 (81.7%)	8 (34.8%)	<0.001

Data are expressed as mean ± standard deviation or n (%). Fisher’s exact test was used for analysis of categorical variables. The Mann–Whitney U test was used for analysis of continuous variables. CSCR: central serous chorioretinopathy; PHS: punctate hyperfluorescent spot; ILM: internal limiting membrane, EZ: ellipsoid zone.

**Table 2:** Presence or absence of cluster PHS and imaging findings in acute or chronic CSCR group.

	PHS (+) Group	PHS (−) Group	*p* Value
Acute CSCR, *n*	32	13	
Filling delay, *n* (%)	29 (90.6%)	12 (92.3%)	1.000
Venous dilation, *n* (%)	30 (93.8%)	10 (76.9%)	0.136
Choroidal vascular hyperpermeability, *n* (%)	26 (81.3%)	8 (61.5%)	0.251
Pachydrusen, *n* (%)	13 (40.6%)	4 (30.8%)	0.737
Chronic CSCR, *n*	31	11	
Filling delay, *n* (%)	26 (83.9%)	9 (81.8%)	1.000
Venous dilation, *n* (%)	28 (90.3%)	8 (72.7%)	0.314
Choroidal vascular hyperpermeability, *n* (%)	25 (80.6%)	6 (54.5%)	0.120
Pachydrusen, *n* (%)	11 (35.5%)	1 (9.1%)	0.133
Acute CSCR unilateral	32	13	
Cluster PHS in CSCR-unaffected fellow eye, *n* (%)	26 (81.3%)	3 (23.1%)	<0.001
Chronic CSCR unilateral	28	10	
Cluster PHS in CSCR-unaffected fellow eye, *n* (%)	23 (82.1%)	5 (50.0%)	0.090

Data are expressed as mean ± standard deviation or n (%). Fisher’s exact test was used for analysis of categorical variables. CSCR: central serous chorioretinopathy; PHS: punctate hyperfluorescent spot.

**Table 3:** Logistic regression analysis for factors associated with PHS cluster in CSCR-affected eyes.

Factors	OR (95% CI)	*p* Value
Univariate		
Male	0.85 (0.25–2.95)	0.798
Age	1.04 (1.00–1.09)	0.045
Systolic blood pressure	1.03 (1.00–1.07)	0.064
Diastolic blood pressure	1.02 (0.99–1.06)	0.241
Visual acuity logMAR	3.23 (0.30–34.85)	0.334
Foveal choroidal thickness	1.00 (1.00–1.01)	0.119
Foveal retinal thickness (ILM-EZ)	1.00 (0.99–1.01)	0.641
Complex CSCR	2.47 (0.80–7.63)	0.116
Chronic CSCR	1.15 (0.45–2.94)	0.778
Filling delay	0.98 (0.24–4.06)	0.980
Venous dilation	3.87 (1.06–14.18)	0.041
Choroidal vascular hyperpermeability	3.04 (1.09–8.48)	0.034
Pachydrusen	2.34 (0.77–7.09)	0.133
Cluster PHS in CSCR-unaffected fellow eye	8.35 (2.84–24.57)	<0.001
Multivariate		
Model 1: adjusted for age and male		
Venous dilation	3.67 (0.95–14.12)	0.059
Choroidal vascular hyperpermeability	2.90 (0.98–8.60)	0.055
Cluster PHS in CSCR-unaffected fellow eye	7.02 (2.21–22.27)	<0.001
Model 2: adjusted for age, male, systolic blood pressure, and foveal choroidal thickness		
Venous dilation	5.53 (1.22–25.12)	0.027
Choroidal vascular hyperpermeability	2.88 (0.84–9.87)	0.093
Cluster PHS in CSCR-unaffected fellow eye	8.47 (2.37–30.26)	0.001
Model 3: adjusted for age, male, systolic blood pressure, foveal choroidal thickness, and complex CSCR		
Venous dilation	5.82 (1.24–27.30)	0.026
Choroidal vascular hyperpermeability	2.93 (0.85–10.11)	0.089
Cluster PHS in CSCR-unaffected fellow eye	9.68 (2.45–38.29)	0.001

PHS: punctate hyperfluorescent spot; CSCR: central serous chorioretinopathy; OR: odds ratio; CI: confidence interval; ILM: internal limiting membrane; EZ: ellipsoid zone.

## Data Availability

Data are available on request from the corresponding author. The data are not publicly available due to privacy and ethical restrictions.

## References

[1] Lam D., Das S., Liu S., Lee V., Lu L., Schachat A.P., Sadda S.R. (2018). Central serous chorioretinopathy. Ryan’s Retina.

[2] Warrow D.J., Hoang Q.V., Freund K.B. (2013). Pachychoroid pigment epitheliopathy. Retina.

[3] Feenstra H.M.A., van Dijk E.H.C., Cheung C.M.G., Ohno-Matsui K., Lai T.Y.Y., Koizumi H., Larsen M., Querques G., Downes S.M., Yzer S. (2024). Central serous chorioretinopathy: An evidence-based treatment guideline. Prog. Retin. Eye Res..

[4] Araki T., Ishikawa H., Iwahashi C., Niki M., Mitamura Y., Sugimoto M., Kondo M., Kinoshita T., Nishi T., Ueda T. (2019). Central serous chorioretinopathy with and without steroids: A multicenter survey. PLoS ONE.

[5] Chhablani J., Cohen F.B. (2020). Central Serous Chorioretinopathy International Group. Multimodal imaging based central serous chorioretinopathy classification. Ophthalmol. Retina.

[6] Scheider A., Nasemann J.E., Lund O.E. (1993). Fluorescein and indocyanine green angiographies of central serous choroidopathy by scanning laser ophthalmoscopy. Am. J. Ophthalmol..

[7] Prunte C., Flammer J. (1996). Choroidal capillary and venous congestion in central serous chorioretinopathy. Am. J. Ophthalmol..

[8] Iida T., Kishi S., Hagimura N., Shimizu K. (1999). Persistent and bilateral choroidal vascular abnormalities in central serous chorioretinopathy. Retina.

[9] Pang C.E., Shah V.P., Sarraf D., Freund K.B. (2014). Ultra-widefield imaging with autofluorescence and indocyanine green angiography in central serous chorioretinopathy. Am. J. Ophthalmol..

[10] Menchini U., Virgili G., Lanzetta P., Ferrari E. (1997). Indocyanine green angiography in central serous chorioretinopathy. ICG angiography in CSC. Int. Ophthalmol..

[11] Tsujikawa A., Ojima Y., Yamashiro K., Ooto S., Tamura H., Nakagawa S., Yoshimura N. (2010). Punctate hyperfluorescent spots associated with central serous chorioretinopathy as seen on indocyanine green angiography. Retina.

[12] Cheng C.M.G., Lee W.K.L., Koizumi H., Dansingani K., Lai T.Y.Y., Freund K.B. (2019). Pachychoroid disease. Eye.

[13] Park S.J., Kim B.H., Park K.H., Woo S.J. (2014). Punctate hyperfluorescence spot as a common choroidopathy of central serous chorioretinopathy and polypoidal choroidal vasculopathy. Am. J. Ophthalmol..

[14] Kim H., Lee J.H., Kwon K.Y., Byeon S.H., Lee S.C., Lee C.S. (2015). Punctate hyperfluorescent spots associated with polypoidal choroidal vasculopathy on indocyanine green angiography. Ophthalmic Surg. Lasers Imaging Retin..

[15] Kim J.H., Chang Y.S., Lee T.G., Kim C.G. (2015). Choroidal vascular hyperpermeability and punctate hyperfluorescent spot in choroidal neovascularization. Investig. Ophthalmol. Vis. Sci..

[16] Kang H.G., Han J.Y., Kim M., Byeon S.H., Kim S.S., Koh H.J., Lee C.S. (2021). Pachydrusen, choroidal vascular hyperpermeability, and punctate hyperfluorescent spots. Graefe’s Arch. Clin. Exp. Ophthalmol..

[17] Matsumoto H., Mukai R., Morimoto M., Tokui S., Kishi S., Akiyama H. (2019). Clinical characteristic of pachydrusen in central serous chorioretinopathy. Graefe’s Arch. Clin. Exp. Ophthalmol..

[18] Spaide R.F. (2018). Disease expression in nonexudative age-related macular degeneration varies with choroidal thickness. Retina.

[19] Koo T.K., Li M.Y. (2016). A Guideline of Selecting and Reporting Intraclass Correlation Coefficients for Reliability Research. J. Chiropr. Med..

[20] Landis J.R., Koch G.C. (1977). The measurement of observer agreement for categorical data. Biometrics.

[21] Kamao H., Goto K., Date Y., Hiraki R., Mizukawa K., Miki A. (2024). Clinical characteristics of punctate hyperfluorescence spots in the fellow eye of patients with unilateral macular neovascularization with no drusen. J. Clin. Med..

[22] Shinojima A., Mehanna C., Lavia C.A., Gaudric A., Tadayoni R., Bousquet E. (2020). Central serous chorioretinopathy: Risk factors for serous retinal detachment in fellow eyes. Br. J. Ophthalmol..

[23] Jirarattanasopa P., Ooto S., Tsujikawa A., Yamashiro K., Hangai M., Hirata M., Matsumoto A., Yoshimura N. (2012). Assessment of Macular Choroidal Thickness by Optical Coherence Tomography and Angiographic Changes in Central Serous Chorioretinopathy. Ophthalmology.

[24] Spaide R.F., Hall L., Haas A., Campeas L., Yannuzzi L.A., Fisher Y.L., Guyer D.R., Slakter J.S., Sorenson J.A., Orlock D.A. (1996). Indocyanine green videoangiography of older patients with central serous chorioretinopathy. Retina.

[25] Bousquet E., Provost J., Zola M., Spaide R.F., Mehanna C., Behar-Cohen F. (2021). Mid-Phase Hyperfluorescent Plaques Seen on Indocyanine Green Angiography in Patients with Central Serous Chorioretinopathy. J. Clin. Med..

[26] Kawakami S., Sasaki M., Wakabayashi Y., Mizusawa T., Mori H., Goto H., Yasukawa T. Clinical significance of punctate hyperfluorescent spots observed on indocyanine green angiography in eyes with central serous chorioretinopathy. *Euretina Congress* 2024 Abstracts. https://euretina.org/barcelona-2024/abstracts/.

